# Comparing SOFA scores of ICU patients in a low income national referral hospital

**DOI:** 10.1186/2197-425X-3-S1-A337

**Published:** 2015-10-01

**Authors:** C Sendagire, D Obua, J Nakibuuka, J Ejoku, A Kwizera

**Affiliations:** Department of Anaesthesia, Makerere University College of Health Sciences, Kampala, Uganda; Department of Medicine, Mulago National Referral Hospital, Kampala, Uganda; Department of Anaesthesia, Mulago National Referral Hospital, Kampala, Uganda

## Introduction

Sub-Saharan Africa has a significantly growing burden of critical illness on account of high prevalence of sepsis, HIV, trauma and obstetric complications. With scarce data on organ dysfunction in low income countries we analyzed the latter by comparing SOFA scores between survivors and non-survivors. We replaced the Pa02/Fi02 ratio with SP02/Fi02 ratio.

## Objectives

To compare SOFA scores between ICU survivors and non-survivors in Mulago National Referral Hospital general intensive care unit.

## Methods

We performed a prospective observational study in Mulago general ICU on patients above 12years. We excluded postoperative patients admitted for low risk monitoring or less than 24hours. The worst SOFA scores were calculated at admission and 48 hours, including the difference thereof. Patients were then followed up to discharge or death.

## Results

135 patients were consecutively enrolled from February 2014 to January 2015, 17 were excluded. Interim analysis was done on 118 patients; the median age 34 years, 57.6% male and the overall ICU mortality 47.5%. Median survival time was 12 days with (95% CI 6.38-17.62).

Comparing the mSOFA score means, the non-survivors vs. survivors; the initial mSOFA (7.7 vs. 5.5; p = 0.007), mean mSOFA (8.1 vs. 4.7; p = 0.00001), and highest mSOFA (9.4 vs. 5.8; p = 0.00001). We found that in survivors the delta mSOFA decreased by 2.7(1.7), it increased by 1 (3.1) after 48hours in non-survivors. On admission, only the SOFA-RS and SOFA-CVS means were significantly different between non-survivors vs. survivors; (2 vs. 1; p = 0.007) and (1 vs 0; p = 0.001) respectively. We however found that at 48hours, non-survivors also had a significantly higher SOFA-Renal and SOFA-CNS mean scores than survivors; (3 vs. 2; p = 0.0001) and (2 vs. 1; p = 0.0001) respectively.

## Conclusions

Non-survivors have significantly higher initial, mean, and highest SOFA scores and more number of organ dysfunctions after 48hours.

## Funding

Self-funded as part of post-graduate thesis.
